# From microtissues to macro solutions – The future of scalable and automated cartilage tissue engineering

**DOI:** 10.1016/j.jot.2026.101150

**Published:** 2026-06-08

**Authors:** Daphne M.A. Menssen, Antonia G. Vasilopoulou, Florencia Abinzano, Jasmijn V. Korpershoek, Aylin Kara Özenler, Ioannis Papantoniou, Daniel J. Kelly, Sebastien J.P. Callens, Jos Malda, Keita Ito

**Affiliations:** a Department of Biomedical Engineering, Orthopaedic Biomechanics, Eindhoven University of Technology, Eindhoven, the Netherlands; b Institute for Complex Molecular Systems, Eindhoven University of Technology, Eindhoven, the Netherlands; c Regenerative Medicine Centre Utrecht, Utrecht, the Netherlands; d Department of Orthopaedics, University Medical Centre Utrecht, Utrecht, the Netherlands; e Department of Orthopedic Surgery, Division of Sports Medicine, Mayo Clinic, Rochester, Minneapolis, MN, USA; f Prometheus the Leuven R&D Translational Division of Skeletal Tissue Engineering, KU Leuven, Leuven, Belgium; g Skeletal Biology and Engineering Research Centre, Department of Development and Regeneration, KU Leuven, Leuven, Belgium; h Trinity Centre for Biomedical Engineering, Trinity Biomedical Sciences Institute, Trinity College Dublin, Dublin, Ireland; i Department of Mechanical, Manufacturing and Biomedical Engineering, School of Engineering, Trinity College Dublin, Dublin, Ireland; j Department of Clinical Sciences, Faculty of Veterinary Medicine, Utrecht University, Utrecht, the Netherlands

**Keywords:** Cartilage tissue engineering, Automation, Scalability, Microtissues, GMP-Compliance, Cost-effectiveness

## Abstract

The burden of articular cartilage damage on society continues to rise. Suitable treatment is necessary to reduce pain, improve quality of life, and avoid progression into osteoarthritis. Current regenerative cartilage repair strategies remain limited in clinical practice due to high costs, complex logistics, patient burden, and poor reimbursement. To ensure that treatment innovations effectively reach the patient, the focus during development should be on automated and scalable solutions. A promising concept is using cartilage microtissues as building blocks in a modular approach to create larger cartilage constructs. By enabling controlled microtissue assembly and reproducible, scalable manufacturing, this could lead to the efficient production of tissue-engineered cartilage implants. Several automated, scalable steps have been described in literature, ranging from cell isolation and expansion to microtissue formation and harvesting, and finally to macrotissue assembly and maturation. However, integrating all these steps into a single, cost-effective, and automated process remains a conceptual idea. This review focuses on potential routes and key choices that should be considered in the early design process of future ready-to-implant tissue-engineered cartilage implants. Furthermore, the added value of investigating and implementing automatic quality measures and cleaning protocols, as well as the recommended regulatory checks and cost-effectiveness implications, are discussed.

The translational potential of this article: Leveraging microtissues for cartilage tissue engineering in a scalable, automated, and GMP-compliant manner could address the growing burden of articular cartilage damage. This review shows the translational potential of this approach by focusing on the early implementation of these concepts in the design process of cartilage tissue-engineered solutions, ensuring compatibility and viability within current healthcare systems.

## Graphical abstract

**Figure:**
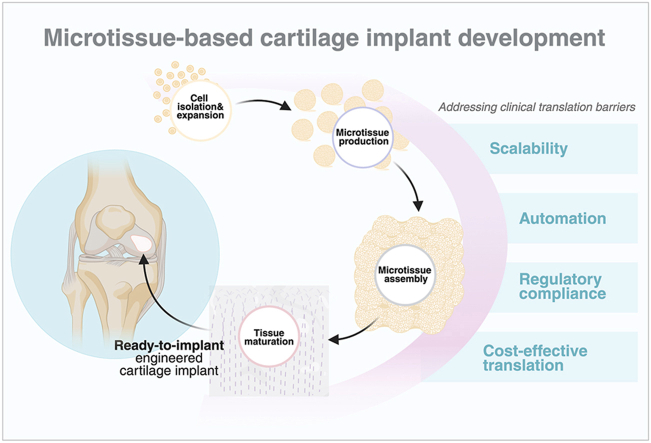

## 1 Introduction

### 1.1 Cartilage defects and current treatment options

Articular cartilage defects in joints such as the knee are common, with defects observed in 60-66% of knees examined under arthroscopy [1]. Articular cartilage is avascular, aneural, and the number of chondrocytes in the tissue is low, contributing to its limited regenerative capacity [2]. Adequate treatment of cartilage defects is important to limit pain, to allow function, improve quality of life, and to avoid progression of joint damage, which could ultimately result in osteoarthritis (OA) and the need for total knee prosthesis.

A common approach to treating cartilage damage involves generating microfractures in the subchondral bone beneath the cartilage defect, allowing stem cell migration from the bone marrow to the defect [3]. However, the formation of fibrocartilage remains a concern, as it breaks down over time due to its inferior mechanical properties compared with those of natural hyaline cartilage [3,4]. An alternative approach is osteochondral autograft transfer surgery (OATS), where osteochondral grafts from a non-load-bearing area of the knee are harvested and placed into the defect [3]. Downsides include donor site morbidity and poor integration with the surrounding cartilage [[5], [6], [7]]. To avoid donor site morbidity, allografts have also been successfully used to treat larger defects. However, this method is still limited by the low availability of fresh donor material and high costs [8,9].

The only regenerative cell therapy for cartilage defects in the clinic to date is autologous chondrocyte implantation (ACI). ACI is a two-step procedure where autologous chondrocytes are harvested, expanded, and used to restore the defect. The implantation of expanded articular chondrocytes (ACs) into a cartilage defect, covered with a periosteal flap (first-generation ACI) or a collagen membrane (second-generation ACI), was shown to promote cartilage repair [10]. However, in these approaches, a mix of fibrocartilage and hyaline cartilage was created due to the dedifferentiation of the ACs during expansion [11]. In an attempt to reverse the dedifferentiation, matrix-assisted chondrocyte implantation (MACI) was introduced (third-generation ACI), where ACs are seeded on a collagenous scaffold, and the construct is implanted as a whole [11,12].

Microtissue-based ACI (third/fourth generation ACI) is also in clinical use and has shown favourable outcomes and hyaline-like cartilage regeneration in defects up to 10 cm^2^ [[12], [13], [14]]. This approach relies on a fully autologous cell product, requiring no additional cell carriers, membranes, or sutures, as the microtissues adhere directly to the subchondral bone [15]. However, immediate weight bearing after surgery is not permitted because the maturation and integration of the microtissues into the host cartilage and subchondral matrix requires several months to years [16]. Consequently, patients face a prolonged rehabilitation period and a delayed return to full activities.

Even though ACI has been in clinical trials for over 30 years, with the Food and Drug Administration (FDA) in the United States approving the first ACI as early as 1997 and the European Medicines Agency (EMA) in 2009, its use in clinical practice remains limited due to patient burden, difficult logistics, high associated costs, and poor, or in some cases, no reimbursement in many countries [[17], [18], [19]].

### 1.2 Microtissue-based cartilage tissue engineering for automated and scalable cartilage implant development

Regenerative medicine and tissue engineering hold great promise for enhancing cartilage repair and enabling the development of off-the-shelf therapeutic products. Given the rising burden of OA in society, it is essential to ensure that these innovations effectively reach the patients. To achieve this, efforts should be focused on developing scalable and automated approaches that make such therapies feasible and appealing from both healthcare providers' and payers’ perspectives. In this context, “automated” refers to the use of programmable closed or semi-closed systems capable of performing process steps with minimal human intervention. Furthermore, while “scalable” refers to the adoption of technologies to increase volumetric or parallelized production throughput, which will result in a larger number of microtissues per batch. In most cases, a certain level of automation, through bioreactors and robotics, is required to achieve or enhance scalability.

**Fig. 1:**
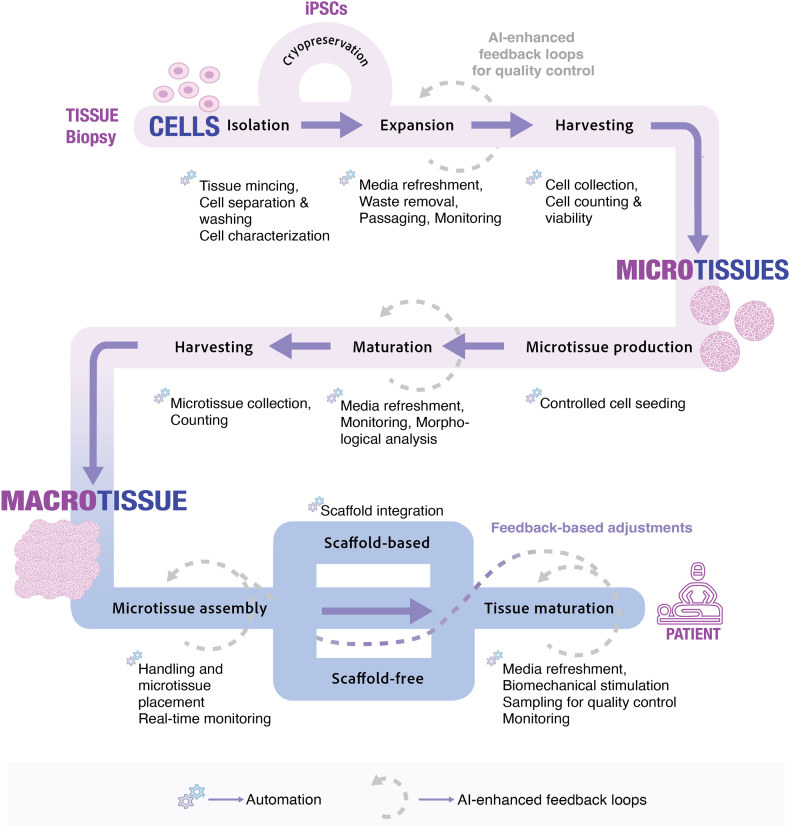
Overview of an envisioned end-to-end workflow for automated development of microtissue-based cartilage implant: from cell isolation to a mature engineered construct. The schematic illustrates the steps requiring automation, and highlights integrated feedback loops for real-time quality control and process optimization. AI, artificial intelligence; iPSCs, induced pluripotent stem cells.

Cartilage microtissues, 3D structures consisting of cells and cartilaginous matrix containing tissue specific properties, can be used as building blocks in a modular approach to create larger cartilage constructs with a high cell density. Compared to conventional single cell culture techniques, microtissues contain cell-matrix and cell-cell interactions which can contribute to chondrogenesis [20,21]. As a result, microtissues are increasingly being used for efficient tissue engineering. Besides supporting a fast and controlled way to create large cartilage constructs, microtissues are also well-suited to be incorporated in novel automatic and scalable manufacturing techniques [22]. However, to date, no fully integrated platform for cartilage tissue engineering automates all stages from cell isolation and proliferation, through microtissue formation, differentiation, potential pre-assembly and construct maturation, and finally, quality control (Fig. 1). A conceptual, automatic, and closed system for osteochondral tissue engineering, which aligns with this idea, called JointPromise, has been described [23]. Other studies merely describe parts of the process in an automated, scalable, or Good Manufacturing Practice (GMP)-compliant manner, referring to manufacturing processes that ensure consistent product quality and control in accordance with regulatory quality standards [24].

Within cartilage tissue engineering, multiple cell sources have been investigated and used in the clinic. However, not all are suitable for high-throughput and large-scale manufacturing approaches. The same challenge applies to the different methods available to create microtissues and assemble them into larger constructs, including the required automatic monitoring and quality control. Here, we review, highlight, and suggest potential routes and choices that should be considered early in the design process of modular large-scale cartilage tissue engineering using microtissues to realize automated, GMP-compliant, and cost-effective processes that meet regulatory standards.

## 2 Scalable and automated cell production for cartilage tissue engineering

Innovations in cartilage tissue engineering rely on a careful selection of an appropriate cell source. Even though many cell sources have been investigated over the years, not all are compatible with large-scale, automatic, and GMP-compliant manufacturing processes. Section 2.1 explores the characteristics, advantages, and challenges of the main cell sources used in cartilage tissue engineering. Building on this, the use of these cell sources in scalable and automatic production processes is discussed in Section 2.2.

### 2.1 Cell sources for cartilage repair

**Fig. 2:**
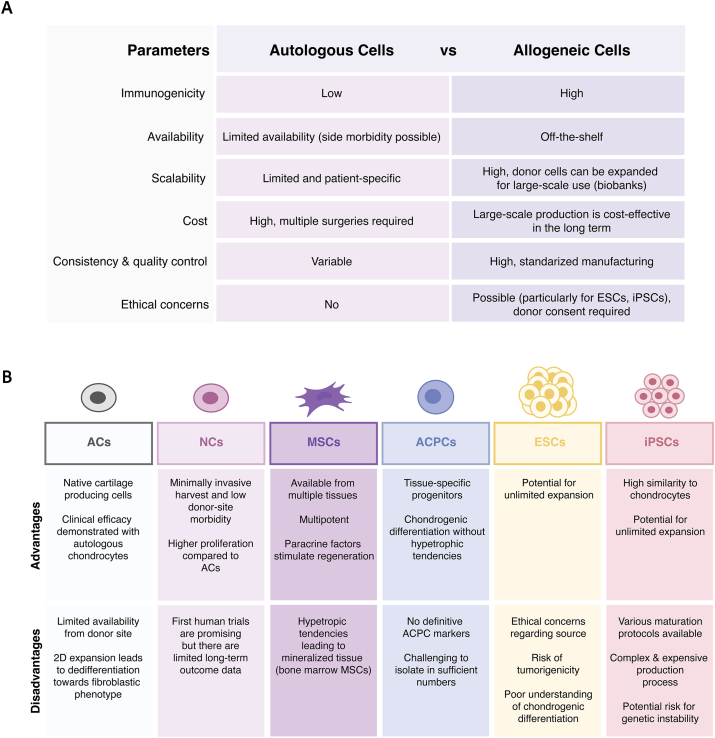
(A) Comparison of autologous and allogeneic cells for efficient cartilage tissue engineering. (B) Summary of the (dis)advantages of different cell types commonly used for cartilage repair. ACPCs, Articular Cartilage Progenitor Cells; ACs, Articular Chondrocytes; ESCs, Embryonic Stem Cells; MSCs, Mesenchymal Stromal Cells; NCs, Nasal Chondrocytes; iPSCs, Induced Pluripotent Stem Cells.

Within cartilage tissue engineering, various cell sources have been explored, each with distinct advantages and disadvantages (Fig. 2). Allogenic cells are inherently more scalable, as they enable the production of large quantities of cells from optimal donors, creating an off-the-shelf product and allowing for a single-stage surgical procedure. However, the success of their translation largely depends on their safety profiles and efficacy in repairing articular cartilage. On the other hand, autologous cells, such as ACs, have already been in use in the clinic for the past decades and show promising results [25]. Since they are patient-derived cells, they pose a very low risk for immune rejection. However, autologous ACs remain limited by two-step procedures, diminished scalability, and higher costs.

Besides the gold-standard use of autologous ACs, chondrocytes harvested from a patient's nasal septum tissue have also been explored for treating knee cartilage defects (NCs, nasal chondrocytes) [26]. Their harvest is minimally invasive, associated with low donor-site morbidity, and does not cause any relevant discomfort to the patient [27]. *In vitro* studies showed that expanded NCs exhibit superior and more reproducible chondrogenic capacity compared to ACs [28,29]. First-in-human studies demonstrated the safety and efficacy of *in vitro*-matured NC-based tissue-engineered cartilage for focal defects (2-8 cm^2^). Those findings further support the clinical relevance of engineered mature tissues for cartilage repair [30,31]. The long-term stability of these engineered tissues needs to be further evaluated.

Mesenchymal stromal cells (MSCs) are multipotent cells that can be differentiated towards the chondrogenic lineage. They are expandable *in vitro*, their paracrine factors stimulate tissue regeneration, and they have anti-inflammatory properties, which contribute to reducing joint inflammation and facilitating tissue repair in OA conditions [[32], [33], [34]]. MSCs can be obtained from several sites, with the most common being bone marrow, though their collection is relatively invasive. Other sources, such as amniotic fluid, adipose tissue, umbilical cord blood, and menstrual blood, have gained attention and shown promising results in cartilage repair [33,34]. For cartilage repair, both autologous and allogeneic MSCs have been used [35,36]. Downsides of using MSCs for cartilage repair include the formation of fibrocartilage and the frequent hypertrophic differentiation of chondrogenically primed MSCs, where they create a mineralized tissue [37,38]. However, the chondro-inductive properties of allogeneic MSCs have been well-demonstrated in human clinical trials, where donor MSCs are combined with recycled autologous chondrons, resulting in enhanced cartilage regeneration in focal defects [32].

Articular chondroprogenitor cells (ACPCs), present in the superficial layer of cartilage, are an attractive cell source in cartilage engineering, with previous studies showing promising results for cartilage repair both *in vitro* [[39], [40], [41], [42]] and *in vivo* [[43], [44], [45]]. ACPCs are capable of extensive proliferation while maintaining the ability to produce a qualitatively better hyaline-like cartilage matrix, rich in type II collagen and glycosaminoglycans (GAGs), compared to both bone marrow- or adipose-derived MSCs (ADSCs) [46,47]. In addition, they maintain their chondrogenic potential during expansion up to 30 population doublings, and they do not undergo hypertrophic differentiation, thus overcoming major challenges in cartilage engineering [48]. Similar to using autologous ACs, using autologous ACPCs will still require a lengthy two-step procedure, where ACPCs, even less plentiful than ACs, need to be harvested from the patient and extensively expanded to reach a sufficient number of cells, making scale-up challenging [49]. The expansion of allogeneic ACPCs in a GMP-compliant manner has already been demonstrated [50], and their use for cartilage repair has been investigated in large animal models [44]. However, allogeneic ACPC-laden constructs showed varying repair outcomes *in vivo*, with no signs of hypertrophic differentiation, yet no satisfactory regeneration of the articular cartilage tissue [45,51].

Overall, to overcome drawbacks related to the use of autologous cells, an off-the-shelf cell source could offer a viable and scalable solution. Therefore, there has been a growing interest in using allogenic pluripotent stem cells. For example, embryonic stem cells (ESCs) can be expanded unlimited, creating a sustainable cell source, while they have also shown good chondrogenic properties upon stimulation [38]. However, risks include tumorigenicity as well as a poor understanding of the process of chondrogenic differentiation, making clinical application difficult [52].

Another promising, off-the-shelf cell source is the induced pluripotent stem cell (iPSC), which can be differentiated towards chondrocytes and used for cartilage regeneration. Encouraging results have been shown in *in vitro* studies, where cartilaginous microtissues were produced using iPSCs [[53], [54], [55]]. Furthermore, *in vivo* animal studies showed successful implantation of iPSC-derived cartilage constructs into cartilage defects of immunodeficient rats [56] and monkeys [57], resulting in good hyaline cartilage repair tissue. To date, multiple differentiation protocols have been described for differentiating iPSCs into induced chondrocytes (iCHOs) that pass through various intermediate cell types, such as induced mesenchymal stromal cells (iMSCs) [55], expandable limb bud mesenchymal (ExpLBM) cells [56,58], and induced chondroprogenitor cells (iCPCs) [53,54,59]. Demonstrating the clinical efficacy and safety of these protocols and reaching consensus on standardized methodologies are essential. Finally, a key challenge in using iPSC lines is their genomic instability, which can occur at any processing stage [60,61].

### 2.2 Automated, GMP-compliant, and scalable cell expansion

Several strategies for large-scale, automated, and GMP-compliant cell expansion have been reported in literature or implemented commercially, particularly within the fields of cell therapy and regenerative medicine. Such strategies or concepts can be employed to obtain sufficient cell numbers for large-scale cartilage microtissue production. The choice of culture system is strongly influenced by the cell type and source, as well as by their growth characteristics under specific conditions. Generally, the cell sources for cartilage repair, as discussed in Section 2.1, are adherent. However, suspension cultures are inherently more scalable compared to automated adherent static cultures, which are limited by surface area. For cartilage engineering approaches, therefore, suspension cultures require either continuous agitation to enable aggregate formation, if the cell type allows, or the addition of matrices or microcarrier components to support cell adhesion. In line with this, several studies have explored agitation suspension cultures for expansion of different cell types used for cartilage repair, including ACs, MSCs, and iPSCs.

ACs tend to dedifferentiate when expanded in a monolayer and also do not grow as single cells in suspension cultures. To enable the scalable expansion of ACs while maintaining a chondrogenic phenotype, growing the cells in or on 3D structures in suspension cultures provides a solution. For example, expanding ACs in 3D aggregates in suspension cultures led to high population doublings with cells producing a cartilaginous matrix [62]. To boost the expansion of ACs in microtissues even more, stimulating factors for aggregation and proliferation have been added, for example, porcine notochordal cell-derived matrix [63]. However, such additives with xenogeneic origin and composition variability face regulatory challenges and restricted clinical applicability [64]. Finally, besides increased scalability, agitated suspension cultures offer advantages including reduced costs, decreased reliance on disposables, and faster culture times [[65], [66], [67]]. For example, it was shown that using a 0.5L agitated suspension culture for iPSC expansion in aggregates results in a 100-fold increase, compared to a 19-fold increase in an adherent 2D culture, which reduces the costs by almost 6-fold [68].

A different approach involves the addition of microcarriers in these dynamic bioreactor systems where the adherent cells can attach to and expand while maintaining chondrogenic properties [69,70]. As an example of the enhanced scalability achievable with these systems, a rotating bioreactor with gelatin methacryloyl (GelMA) microcarriers has been shown to achieve a 16-fold increase in the expansion of adherent cells in just eight days, including the advantage that the microcarriers are quickly dissolved, simplifying cell harvesting [71]. As a next step, a closed, automated bioreactor incorporating dissolvable microcarriers has been recently described, enabling the cell expansion in scalable expansion bags with automatic medium changes [72]. Advantages of such a system include the decreased handling and removal of human errors, therefore reducing the contamination risks and enabling controlled cell expansion [67,72].

While suspension cultures offer greater scalability, automated and closed adherent static techniques have also been widely investigated and commercialised. There are several technologies that have been introduced by manufacturers and used for large-scale production of clinical grade MSCs, such as CliniMACS Prodigy® (Miltenyi Biotec), Cocoon® Platform (Lonza), and Quantum® Cell Expansion System (Terumo BCT). All these systems enable automatic cell expansion, medium refreshment, and harvesting under closed and controlled conditions [73]. For iPSC production and expansion, another system known as “StemCellFactory” has been introduced. This system automates the entire process, from fibroblast expansion and reprogramming into iPSCs to their subsequent expansion in adherent well plates [74]. Besides production and expansion, cell differentiation can also be automated in plates, as seen in the AutoCRAT system [75]. Despite these advancements, such platforms remain very complex and rely on expensive consumables, making them a costly manufacturing option overall, particularly for autologous cells that are typically produced in smaller batches.

Apart from the cell type's growth requirements, the cell origin should be also taken into consideration when designing a culture system for automated cell manufacturing. For instance, the use of autologous cells introduces variability in both quality and quantity, creating the need for automated platforms that are not only able to isolate the cells from the patient's tissue and expand them, but dynamically adapt to a broad range of cellular behaviours [76]. A system for automatic and GMP-compliant isolation of ACs from a cartilage biopsy and subsequent adherent cell culture has been proposed [67]. This system focuses on reducing contamination risks and creating a reliable, consistent product of engineered cartilage tissue by using robot arms to mince cartilage and isolate, seed, and culture cells with automated medium changes and cleaning [67].

## 3 Scalable cartilage tissue engineering using microtissues

Following cell expansion and harvesting, single cells must transition to a 3D microenvironment that promotes cell-cell interactions and supports microtissue formation. This process is initiated by intercellular adhesion molecules, such as N-cadherin, along with cytoskeleton integrins and proteins, which drive cell-cell adhesion, aggregation and early compaction [77]. Once intercellular adhesion is established, the cells begin producing extracellular matrix (ECM), which binds to the cytoskeleton via anchoring proteins, such as integrins [77,78]. This interaction of cells with the ECM fibres further contributes to structural organization and provides both mechanical and biochemical cues that guide early phenotypic maturation [78]. Over time, increased cell adhesion and matrix deposition lead to the formation of compact, spherical, and mechanically stable microtissues. Moving to the next step in the workflow for microtissue-based cartilage implant production, Section 3.1 focuses on the use of microtissues as building blocks in cartilage engineering, and Section 3.2 discusses their automated and scalable production across different 3D environments.

### 3.1 Microtissues as building blocks for cartilage engineering

Engineering cartilage grafts from single cells to repair large-scale defects is challenging, as it requires high cell numbers, often leading to diffusion limitations resulting in nutrient and growth factor gradients impacting viability and the phenotypic homogeneity of the resulting tissue [79,80]. This has motivated the increased interest for using microtissues, typically possessing properties below diffusion limitation thresholds, that have been engineered in controlled conditions, as building block units to engineer large-scale cartilage implants with a predefined shape [81]. The microtissues are often referred to as spheroids [12,[82], [83], [84]], cellular aggregates [85,86], or organoids [63,64,87,88]. While these terms are used interchangeably in the literature, no clear consensus exists regarding their definition, and the existing definitions in the field of cartilage engineering are non-specific and overlapping. Therefore, we adopt the term cartilage microtissue throughout this review for consistency. Nevertheless, they have been previously defined as follows: aggregates are clusters of cells, spheroids are spherical, compact clusters of cells with a specific phenotype, and organoids are formed from cells that self-organize into more complex, tissue-like structures with autonomous biological functionality [79].

Cartilage microtissues are typically formed through self-assembly in a non-adhesive environment such as microwells, hanging drop systems, or agitating bioreactors. This process resembles embryonic development, where cells aggregate without any external influence [89]. Additionally, the 3D architecture of microtissues provides an environment that facilitates native-like spatial distribution of cells, in contrast to 2D monolayers or single-cell suspensions [90]. Such an environment supports enhanced cell-cell and cell-matrix interactions, protein secretion, ECM deposition, stable morphology, polarization, and physiological metabolic function [89]. For example, maturing MSCs in microtissues overcomes challenges commonly observed with chondrogenically differentiating single MSCs, such as mineralization and restricted nutrient diffusion [80,91]. While microtissues often exhibit high cell numbers compared to native cartilage, the cells in microtissues are capable of producing a significant amount of cartilaginous matrix [21].

Numerous studies have shown that microtissues can be successfully assembled and fused into millimetre- or even centimetre-scale constructs for cartilage and bone repair [[92], [93], [94], [95], [96], [97]]. Their use leads to controlled and improved neo-tissue shape fidelity [[97], [98], [99], [100]]. Microtissue fusion is often explained by the differential adhesion hypothesis (DAH), which suggests that the liquid-like behaviour of the microtissues allows them to adhere to one another via N-cadherin-mediated junctions and gradually merge into larger structures [101]. Fusion efficiency is further affected by cytoskeletal dynamics, as frequent changes in cytoskeletal organization in active cells increases tissue fluidity and improves fusion [102]. In addition, biological factors such as cell type and culture conditions influence fusion behaviour. It was recently shown *in vitro* that AC microtissues only fused in the medium supplemented with transforming growth factor-beta (TGF-β) and/or L-ascorbic acid [103], whereas the fusion capacity of MSC microtissues appears largely independent of growth factor stimulation regimens [104].

A commonly encountered challenge in microtissue-based implant development is the insufficient remodelling after microtissue fusion, which leads to persistent spheroid architectures and incomplete remodelling into a unified tissue [80,[105], [106], [107]]. Several solutions are investigated and could be combined to improve this challenge. For example, using a higher number of smaller microtissues has been proven to create a more hyaline-like cartilage structure with homogeneous matrix distribution [81]. Smaller and spherical microtissues are known to have a higher stiffness and to fuse faster [21,108]. In smaller microtissues, molecules can diffuse better, leading to a more homogeneous oxygen tension throughout the microtissue, which is beneficial for a more controlled chondrogenic differentiation in hypoxic culture conditions [100,[109], [110], [111]]. Furthermore, spherical geometries are better at filling the voids between each other [112]. Finally, besides their size, the maturation state of the microtissues also affects their fusion capacity and remodelling potential, with less mature spheroids generally demonstrating superior fusion [21].

In addition to microtissue-related parameters, biomaterial properties play a key role in regulating both fusion and post-fusion remodelling. In the presence of a hydrogel, the fusion phenomenon depends on the inter-spheroid distance and cell migration potential, both of which are largely affected by hydrogel characteristics, such as viscoelasticity and degradation rate [63,[113], [114], [115]]. Rapidly degrading hydrogels permit dynamic matrix remodelling, enhance nutrient and oxygen diffusion throughout the construct, and thereby support more uniform tissue maturation [114]. Likewise, low stiffness hydrogels with fast stress relaxation behaviour facilitate cell migration out of the individual microtissues and promote their reorganization into a unified tissue [116]. For cartilage microtissues specifically, more viscous environments were shown to promote ECM deposition [63,116]. However, in cartilage tissue engineering, the mechanical properties of the hydrogel must be carefully balanced to support chondrogenic differentiation. Excessively stiff hydrogels can lead to stress shielding and promote fibroblastic phenotype, whereas too soft gels may induce undesirable adipogenic differentiation in certain cell types, such as MSCs. Interestingly, it was recently shown that fine-tuning the mechanical properties of support hydrogel baths in microtissue bioprinting platforms, enables the precise control over the physical constrains applied to the microtissues. This regulates their phenotype, fusion, and ECM organization [117]. In particular, increased bath stiffness was shown to enhance collagen fibre alignment. Other biomaterials to provide structural support, including biodegradable polymer scaffolds, can also affect microtissue fusion through their architecture, pore size, and degradation behaviour. For example, polycaprolactone (PCL) bucket-shaped microfibre scaffolds with lateral pores between the fibres increase pore interconnectivity, thereby facilitating microtissue interconnection and fusion [118].

### 3.2 Microtissue production

Besides the automated and scalable expansion of cells as described in Section 2.2, the automation of microtissue formation and culture is also of great significance in realizing a cost-effective tissue production process (Table 1).

**Table 1:** An overview of studies highlighting scalable, automatic, or GMP-compliant parts of the cartilage tissue engineering process, indicating the specific microtissue studied.

	Reference	Concept	Scalable	Automatic	GMP-compliance
**Cell expansion and maturation/differentiation**	Cuesta-Gomez et al. (2023) [68]	Agitated suspension culture for the expansion of iPSCs.	X		X
	Haeusner et al. (2021) [67]	Conceptual design of an automated platform for cell isolation from patient biopsies and cell culture.	X	X	X
	Rogers et al. (2021) [71]	Agitated suspension culture for the expansion of iMSCs with GelMA microcarriers.	X		
	Das et al. (2019) [72]	Agitated suspension culture for MSC expansion with dissolvable microcarriers	X	X	X
	Lawson et al. (2017) [119]	Agitated suspension culture for the expansion of MSCs with microcarriers.	X		
	Herbst et al. (2023) [75]	Automated iPSC expansion and differentiation into iCHOs and iMSCs in plates.	X	X	X
	Fujisawa et al. (2023) [56]	Agitated suspension culture for chondrogenic differentiation of ExpLBM cells.	X		
	Elanzew et al. (2020) [74]	Automatic iPSC production and expansion in well plates.	X	X	
**Microtissue production**	Crispim et al. (2021) [63]	Agitated suspension culture for cartilaginous organoids formation from ACs and a porcine matrix additive.	X		
	Fujisawa et al. (2023) [56]	Agitated suspension culture for cartilaginous particle formation.	X		
	Zieger et al. (2024) [120]	Automated tumour spheroid formation in hanging droplets.	X	X	
	Fürsatz et al. (2021) [121]	Autonomous spheroid formation by using compartmentation of wells plate surfaces.	X		
	Hanaki et al. (2024) [122]	Autonomous chondrocyte particle formation by using CAT plates with highly adherent spots.	X		X
	Futrega et al. (2015) [112]	Cartilage microtissue production in microwells using a mesh to allow for rapid medium changes without losing microtissues.	X		
	Decoene et al. (2024) [123]	Automated medium changes with cartilaginous microtissues in microwells.	X	X	
	Krieger et al. (2022) [23]	Conceptual design of automated platform from culturing cells and aggregates to assembling osteochondral constructs using microtissues.	X	X	X
**Microtissue assembly**	Le et al. (2025) [124]	Assembly of spheroids in a layer controlled by a mesh.	X		
	Spagnuolo et al. (2025) [21]	Assembly of cartilage microtissues by seeding them in an agarose well of 10 mm in diameter.	X		
	Burdis et al. (2022) [81]	Assembly of microtissues with different phenotypes spatially in a 3D framework.	X		
	Hall et al. (2023) [125]	Assembly of cartilaginous microspheroids in a melt electro written scaffold by seeding.	X		
	Spagnuolo et al. (2026) [117]	Extrusion printing of microtissues in mechanically tunable support baths.	X		
	Kronemberger et al. (2024) [114]	Assembly of cartilage microtissues in a degrading hydrogel using extrusion-based 3D bioprinting.	X		
	Mekhileri et al. (2018) [100]	Automated picking and assembly of microtissues using a 3D bioprinter in layers or in a scaffold.	X	X	
	Zieger et al. (2024) [120]	Automated picking and deposition of spheroids and organoids from a reservoir with a camera and size threshold-based image segmentation.	X	X	
	Grexa et al. (2021) [126]	Automated picking and deposition of spheroids based on microscopy and deep learning.		X	
	Nakamura et al. (2021) [96]	Automatic assembly of iPSC-derived spheroids using the Kenzan method.	X	X	
	Nonaka et al. (2025) [127]	Automatic assembly of ADSC-derived cartilage spheroids using the Kenzan method.	X	X	
	Aguilar et al. (2019) [128]	Bioprinting of MSC spheroids with the Kenzan method.	X		
	Hall et al. (2024) [129]	Laser-assisted bioprinting of cartilaginous spheroids.	X		
**Maturation cartilage construct**	Crawford et al. (2012) [130]	Bioreactor for culturing cartilaginous constructs under varying hydrostatic pressures.		X	
	Gabetti et al. (2022) [131]	3D-printed automatic perfusion bioreactor for bone tissue.		X	
	Meinert et al. (2017) [132]	Bioreactor for biaxial mechanical loading of cartilage constructs.		X	
	Smith et al. (2018) [133]	3D-printed bioreactor for tissue culture and perfusion, possibly for mechanical stimulation.	X		
**Quality checks**	Schwedhelm et al. (2019) [134]	Automatic real-time microscopic imaging of iPSC aggregation in suspension culture.	X	X	
	Herbst et al. (2023) [75]	Automatic testing for mycoplasma and endotoxin, fingerprinting assays, and glucose and lactate checks during iPSC expansion and differentiation.	X	X	X
	Decoene et al. (2024) [123]	Automated image analysis for morphology and placement of cartilaginous microtissues in microwells.	X	X	
	Krieger et al. (2023) [135]	Automated and high-speed imaging and processing to evaluate sizes and more of stem cell spheroids in wells.	X	X	X
	Beghin et al. (2022) [136]	Automated live 3D imaging and analysis of the phenotype of cells in organoids in wells.	X	X	
	Haeusner et al. (2021) [67]	Conceptual design of automated cleaning by spraying decontaminating agents and drying with sterilized air.	X	X	X
	Benning et al. (2020) [137]	Automated analysis of cell viability in spheroids using a deep learning image algorithm.	X	X	

Spinner flasks and other agitation-based bioreactors enable the large-scale spherical microtissue production in a dynamic environment that promotes nutrient and oxygen delivery and removal of metabolic waste [138]. Such systems have been widely used to generate chondrogenic microtissues from various cell sources and to promote the re-differentiation of dedifferentiated chondrocytes [63,85,139,140]. To enhance scalability, cartilaginous microtissues were successfully produced in spinner flasks using primary bovine and human ACs with a matrix additive or human ACPCs with Bone Morphogenic Protein 9 (BMP-9) [63,64,87]. Moreover, spinner flasks were also used to create plentiful cartilaginous microtissues efficiently from iPSCs within six weeks [56]. Even though these methods are not automated, and the control over factors such as temperature and oxygen is still lacking [119], medium changes are fast and easy, and there is potential to translate this to existing concepts of automated agitated bioreactors [71,72]. A drawback of this method is the wide range in the size of the microtissues produced [56]. However, they can be filtered to gain a more uniform size range; yet this process inevitably results in the loss of some microtissues.

While suspension cultures are valued for their simplicity and efficiency, microwell- or plate-based static automatic techniques to create cartilaginous microtissues are also explored. These systems offer the advantage of producing microtissues with high uniformity in size and shape, along with enhanced reproducibility, which is necessary for GMP manufacturing [141]. Commercially available microwell plates (e.g., Elplasia plates, Corning, and AggreWell plates, StemCells) have good optical properties for automated imaging and real-time monitoring, whereas tissue culture flasks with microwells (e.g., Elplasia 12K, Corning) enable large-scale microtissue production. While such systems offer high reproducibility, the potentially increased costs and feasibility of implementing them at an industrial scale must also be carefully considered.

Cell seeding in static culture systems can be automated using robotic systems. For instance, an automated robotic system (named ReBiA) has been used for creating 3D epithelial tissue [142]. This system, equipped with a dual-arm robot, enables fully automated 2D cell expansion and seeding of cells in wells for self-aggregation in culture inserts or other 3D environments. Such a liquid handling robotic arm could be combined with an approach for creating cartilaginous microtissues, as shown in a recent study [122]. A remaining challenge when using static microwell culture systems is microtissue displacement during culture or medium refreshments. Possible solutions include adding a membrane with the pore size of single cells over microwells to prevent displacement during automated handling [112], or using deeper microwells [124]. An automated system for medium changes of cartilaginous microtissues in microwell plates has also been developed, showing that increased speeds for aspirating and dispensing medium do not affect the microtissues, allowing for fast processing [123]. The concept of the JointPromise platform combines these ideas by being able to handle diverse microwell shapes for cartilage microtissue production [23].

A simple and low-cost way to generate microtissues is the hanging drop method, where small droplets of cell suspension are dispensed on a non-adhesive substrate that is then inverted. Gravitational forces drive the accumulation of cells at the droplet's lowest point, promoting their self-assembly into microtissues [143]. Efforts have been made to automate this concept by building a liquid-handling platform with integrated cell suspension mixing and a non-contact piezoelectric dispenser [120]. Moreover, in this system, the microtissues could be easily recovered from the droplets with a single centrifugation step. Commercial hanging drop plates (e.g., Akura, InSphero) are also compatible with automated liquid handling systems and allow for easy transfer to spheroid culture plates. Finally, microfluidic hanging drop systems have been developed to enable automated medium refreshments and extend the cultivation time of the microtissues in the droplets [144]. However, the hanging drop method yields smaller microtissues with fewer cells than other microtissue production systems [121].

## 4 Automated and scalable microtissue assembly for cartilage implant generation

### 4.1 Microtissue assembly

To assemble larger tissue structures from microtissues, multiple methods are widely investigated and extensively reviewed [79,145]. Generally, they can be divided in top-down and bottom-up approaches. Top-down approaches rely on scaffolds where single cells can be seeded on and that could provide immediate stiffness, patterning, and good regulation of starting conditions [146]. For example, ADSCs seeded on a scaffold with a certain composition of collagen and silk fibroin showed proliferation and differentiation towards chondrogenic cells, demonstrating how scaffolds can regulate cell behaviour [147]. However, top-down approaches are limited by non-uniform cell seeding, restricted possibilities for spatial organization of phenotypically different cells, and a poor reproducibility and controllability during tissue development [148]. Therefore, these approaches are often not suitable for scalable tissue manufacturing.

These limitations can be addressed with bottom-up tissue engineering techniques, where small microtissues serve as modular building blocks. For example, cartilaginous microtissues can be placed together in a confined environment where they fuse and self-assemble into a large-scale cartilage construct [21,149]. Self-assembly is a typically scaffold-free, inherently cell-driven method of generating larger macrotissues through spontaneous microtissue fusion. *In situ* self-assembly of microtissues has shown clinical efficacy in cartilage repair, as demonstrated in recent studies using spheroid-based ACI [12,16]. For *in vitro* self-assembly in controlled culture conditions, low-adhesion materials, such as agarose, are commonly used to fabricate macrowells for microtissue seeding. These non-adhesive wells prevent cell attachment, facilitate microtissue fusion, and provide spatial confinement. This method was used to produce osteochondral implants consisting of genetically distinct populations of cartilage microtissues that were allowed to fuse in a confined space before subcutaneous implantation [106]. Similarly, in another study, cartilage constructs of ⌀10 mm^2^ were successfully created by fusing microtissues in an agarose well [21]. While this method allows for the generation of relatively large cartilage constructs, their limited restriction by the scaffold walls results in shape changes due to cell-mediated contraction [150]. To constrain the uncontrolled fusion and shape changes, microtissue fusion could be performed using scaffold meshes above and below the construct [124]. Nevertheless, the self-assembled cartilage constructs typically lack the native type II collagen organization within the ECM, resulting in suboptimal mechanical properties [81]. However, mechanical stimulation within a bioreactor system or biochemical stimulation approaches could be used during the maturation process of the structure to induce collagen alignment. Recently, it was shown that stimulating fusing cartilage microtissues in a transwell system with TGF-β1 from the bottom resulted in enhanced type II collagen alignment [149]. So, these bottom-up self-assembly methods are simple and relatively easy to scale up, yet they often face challenges in achieving reproducible construct geometry and mechanical stability.

**Fig. 3:**
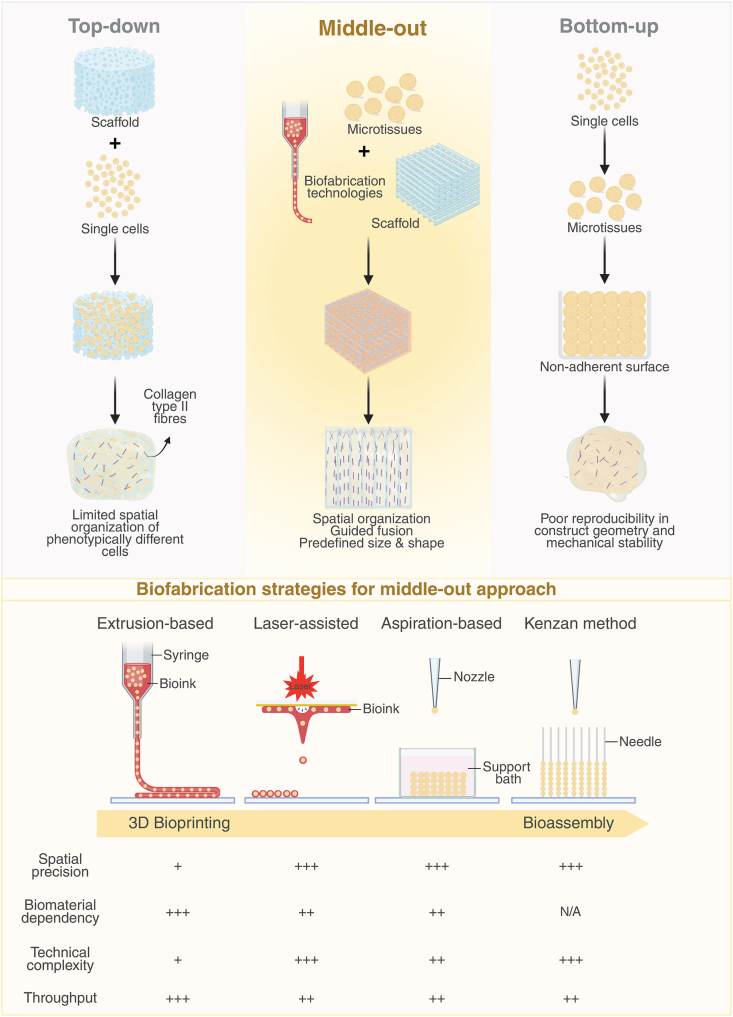
Strategies for microtissue assembly in cartilage tissue engineering. (Top) Schematic illustration of top-down, middle-out, and bottom-up approaches. (Bottom) Overview of four biofabrication techniques used for the assembly of microtissues into larger constructs. The techniques are positioned along a 3D-bioprinting to bioassembly spectrum, and they are compared across spatial precision, biomaterial dependency, technical complexity, and throughput (low: +, moderate: ++, high: +++, and not applicable: N/A). Created with BioRender.

For the precise placement of microtissues, offering greater control over tissue architecture and integration with biomaterials, combinations of top-down and bottom-up approaches, so-called middle-out approaches, have been explored (Fig. 3) [21,114,151]. Incorporating scaffolds provides immediate mechanical strength to the engineered construct and enables the localized delivery of bioactive factors. Specifically, microtissues made from cells with various phenotypes could be spatially placed in such a framework to achieve organization [81,100,152]. These approaches often use 3D-printed polymer scaffolds, for example, melt electrowritten (MEW) fibrous meshes with micrometre-scale pore sizes [81,125,153,154]. Biofabrication strategies have been developed to enable the controlled spatial patterning of microtissues in these scaffolds, a level of organizational control that bulk casting approaches cannot reliably achieve. Therefore, these strategies are promising for guiding fusion and ECM formation, and generating functional constructs with predefined shape and size [81]. The feasibility of bioassembling an osteochondral implant has been demonstrated by spatially placing two populations of phenotypically distinct cartilage microtissues within a 3D polymer scaffold, thereby recreating the zonal cartilage-bone interface [81].

For fully automated biofabrication of cartilage implants, bioprinting and bioassembly systems are powerful tools [155]. In cartilage tissue engineering, several biofabrication strategies have been developed and continue to be explored, including extrusion-based bioprinting, laser-assisted bioprinting, aspiration-assisted bioprinting, and the Kenzan method (Fig. 3) [156]. Firstly, the most commonly used technique is extrusion-based bioprinting, which allows the deposition of spheroid-laden hydrogels, enables the rapid fabrication of macroscale constructs, and offers scalability at relatively low cost and technical complexity, making it the most accessible approach of the above [117,157]. For example, it has been used to place cartilaginous microtissues in an oxidized alginate hydrogel, which quickly degrades, eventually creating a scaffold-free scaled-up cartilage graft [114]. For scaffold-based, middle-out approaches, extrusion-based bioprinting can be converged with MEW for the deposition of cell-laden hydrogels within MEW PCL scaffolds [158,159]. However, this bioprinting method requires a biomaterial-based bioink, consisting of microtissues within a hydrogel, and its spatial resolution is inherently limited by the nozzle diameter [160].

Secondly, Laser-Induced Forward Transfer (LIFT) is a droplet-based bioprinting method that has recently been explored for spheroid patterning [129,161]. Its nozzle-free mechanism prevents clogging and ensures high positional accuracy, making it particularly suitable for arranging spheroids in scaffolds into zonally organized cartilage structures. Additionally, LIFT is compatible with low viscosity bioinks and can be used for the deposition of microtissues suspended in culture medium, reducing the biomaterial dependency compared to extrusion-based approaches [129]. However, its throughput is relatively low, limiting its utility in producing large-scale spheroid cartilage constructs [162]. To address this, efforts towards automation, including integration of computer-aided live imaging analysis for real-time spheroid recognition and targeted transfer, have been reported [129]. Nevertheless, LIFT bioprinting remains a technically demanding approach.

Thirdly, several research groups developed systems for aspiration-assisted bioprinting that integrate robotic manipulation with real-time automated imaging to facilitate the handling and assembly of microtissues into large, organized constructs. The technique can be focused on image-guided robotic pick-and-place systems, such as the CellCelector™ [82] and SpheroidPicker [126]. A similar technology, known as Pick-Flow-Drop, has been developed, where microtissues are imaged and aspirated from a microtissue suspension reservoir (with 98.1% efficiency) and dispensed to precise locations in plates (with 98.4% efficiency) [86]. The aspiration-based approaches are inherently more time-intensive compared to extrusion-based bioprinting, but offer great control over microtissue positioning, comparable to that achieved with LIFT bioprinting [155]. To improve scalability, the HITS-Bio (High-throughput Integrated Tissue Fabrication System for Bioprinting) was recently introduced, where spheroids can be positioned precisely and in a 5-fold faster rate than conventional aspiration-assisted bioprinting [163]. In terms of biomaterial dependency, this approach does not require a hydrogel-based bioink, though a supporting or a sacrificial hydrogel is often incorporated to maintain the microtissue positioning following printing.

Nevertheless, the downside of using biomaterials as scaffolds in middle-out approaches is that some of them could interfere with regeneration and remodelling *in vivo* [106,152,164]. For instance, scaffolds, whether polymer or hydrogel-based, should be biodegradable and have suitable mechanical properties that facilitate matrix turnover rather than inhibiting neo tissue formation and growth [164]. Therefore, a scaffold-free approach may be simpler from a regulatory perspective, as biomaterials approved by regulatory agencies are still limited [123]. Aspiration-assisted bioprinting technologies can also be used to produce scaffold-free constructs [165]. For example, cartilage microtissues, consisting of ADSCs and their chondrogenic ECM, can be aspirated and positioned between pins that are removed after fusion. Next to that, hydrogels can also be used as a temporary support [166]. MSC microtissues have been aspirated and precisely printed on an alginate substrate and subsequently covered with alginate. After fusion, the alginate can be removed with sodium citrate, resulting in a fully scaffold-free construct [167].

Finally, an established bioassembly strategy that results in scaffold-free constructs is the Kenzan method, in which microtissues are placed onto a microneedle array by an automatic robotic system and held in place until they fuse [83,128]. The production of a scaffold-free cartilage graft up to 6 cm^2^ with the Kenzan method has been demonstrated using iPSC-derived microtissues, and with ADSCs microtissues [96,127]. The Kenzan method provides spatial precision comparable to LIFT and aspiration-assisted bioprinting but is limited by the microtissue size and the fixed array of needles [128]. Furthermore, the Kenzan method faces scaling limitations, including the requirement for large numbers of uniform spheroids with proper ECM deposition and the extended fusion time before the construct achieves functional mechanical strength, making it technically complex and costly to implement.

Beyond these well-established microtissue assembly strategies, an emerging technique in the field of biofabrication is cryobioprinting, which combines bioprinting with cryopreservation methods. By enabling the fabrication of off-the-shelf constructs that can be stored, transported, and implanted on demand, cryobioprinting could substantially reduce production timelines and logistical constraints [168,169]. During cryobioprinting, the freezing process allows for control over the construct's porosity and anisotropy by adjusting parameters, such as substrate and printbed temperature. For example, cryoprinting can be combined with electrospinning technologies, enabling the fabrication of a highly porous, multizonal scaffold that better mimics the architecture of native cartilage and supports chondrogenic differentiation [170]. Although still at an early stage of development, cryobioprinting may offer opportunities for more standardized and scalable cartilage implant production when considered early in process design.

### 4.2 Maturation of microtissue-based engineered cartilage constructs

Maturation is an important step in microtissue-based cartilage engineering, where cells remodel the matrix based on mechanical and biochemical cues into mechanically functional tissue. Tissue maturation must occur either *in vivo*, requiring prolonged rehabilitation protocols that delay full weight bearing, as seen in microtissue-based ACI, or *ex vivo* through chondrogenic maturation of the engineered tissue before implantation, followed by an appropriate rehabilitation protocol to ensure good integration. For example, this maturation can be promoted by stimulating the tissue with mechanical loads, growth factors, and low oxygen tension [65,164]. Mechanical stimulation could be achieved by applying cyclic compression, shear stresses, or hydrostatic pressure [171,172]. Neocart was a cartilage tissue engineered implant that was matured in a bioreactor that simulated the joint environment in terms of oxygen tension, hydrostatic pressure, and flow rates [130]. However, Neocart was terminated in 2019 after the end of the phase III randomized controlled superiority trial due to insufficient evidence for improvement compared to microfracture treatment [173]. To fully automate cartilage implant development, adding an automated, standardized, and GMP-compliant method for maturation is desirable, for example, an automatic perfusion bioreactor with uni- or bi-directional flow, applying shear stress on the tissue [131]. In this case, disposable bioreactors could be used to decrease costs and maintain sterility. In addition, for customization, one could consider 3D printing of bioreactors based on patient-specific geometries from image reconstructions [65]. FABRICA is a 3D printed bioreactor designed to be applied for various tissues, that can culture, perfuse, and observe the tissues in real-time [133]. An automatic bioreactor system has been developed that applies direct mechanical stimulation, either uni- or bi-axial shear and compression, to hydrogels loaded with human ACs, which stimulated chondrogenic potential and ECM production [132].

## 5 Automated process monitoring and quality control

**Fig. 4:**
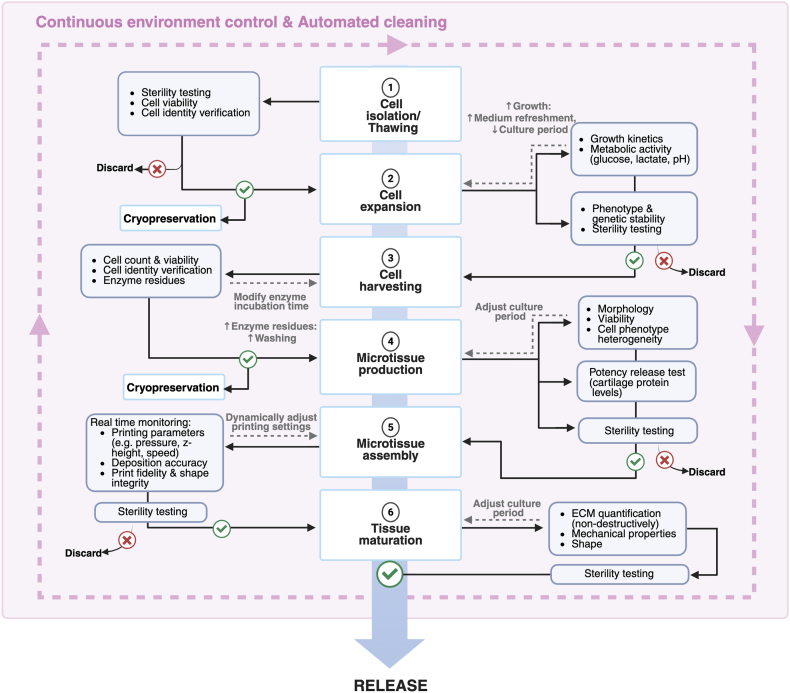
An overview of a potential end-to-end workflow for automatic quality controls and feedback loops for cartilage tissue engineered products. The workflow stretches from cell isolation through tissue maturation, resulting in a cartilage implant. Each steps contains stage-specific quality control measures and feedback loops, resulting in go/no go decisions and enable process adjustments. Together with continuous environment controls and automated cleaning, these quality controls combined support the reproducible and scalable production of cartilage implants. ECM, extracellular matrix. Created with BioRender.

Quality measures, preferably automatic, are important to ensure that the product is consistently manufactured to meet defined quality standards, based on GMP regulations (Fig. 4). For any cell-based product, controlling cell identity and purity, including the presence of off-target cells, microbes, and residual materials from the culture process, is essential. For example, iPSC-derived cells should be monitored for quality before use to confirm the absence of off-target cells [53,65,74,174]. Standardized quality checks on hypertrophic or fibroblastic markers are also required with autologous cells to achieve safety and efficacy, as donor-to-donor variation is unavoidable. For allogeneic cells, safety testing should include analyses of immunogenicity, tumorigenic potential, and genetic stability. Commonly used assays include flow cytometry, quantitative reverse transcription-polymerase chain reaction (qPCR), and karyotype analysis [175]. The system should also be capable of testing for mycoplasma, endotoxin, and other quality measurements, such as cell viability [75].

When moving from cells to microtissues, high-speed microscopy can be built into an automated platform to directly evaluate the quality of microtissues by using computer vision algorithms that can determine size, shape, and empty wells [135,176]. Even though this has been described for both agitated suspension cultures [134] as well as static cultures [123,135], monitoring static cultures without sacrificing microtissues seems to be more accessible. To characterize the cell phenotype heterogeneity in the microtissues, high-speed 3D imaging combined with convolutional neural network-based algorithms for 3D segmentation analysis of cell nuclei and for classifying microtissue morphology can be used [136]. Finally, to ensure product quality and function, a potency release test should be included, such as measurement of cartilage protein expression levels in the microtissues [15].

Integrated quality control and compliance with regulatory agencies, are also essential for bioprinting with microtissues, particularly in extrusion-based systems, which are inherently prone to reproducibility challenges due to their open-loop nature [177]. In such systems, the output is not measured and therefore not used as feedback to adjust the process in real-time. The printing process is highly sensitive to changes in bioink properties and environmental conditions, which can cause variations that might compromise the tissue construct's structure and functionality. Recently, an *in situ* quality monitoring system was developed that allows for real-time monitoring of the print quality with an integrated high-resolution camera and generates visual data records that serve as auditable evidence of the bioprinting process [178]. Similarly, a real-time monitoring strategy was established by integrating a liquid flow sensor as a part of a pneumatically driven extrusion bioprinter. The sensor enabled continuous monitoring and control of the flow rate of the dispensed ink, while a Python-based software tool processed the data in real-time to dynamically adjust the extrusion pressure [179].

After the successful assembly of the microtissues into the larger cartilage constructs, automated and non-invasive methods are needed to evaluate the engineered tissue. Quantification of ECM biomolecules, and particularly GAG content, in engineered constructs with a thickness up to ∼3 mm can be non-invasively monitored with fibre-optic Raman spectroscopy and combined with machine learning (ML) [180,181]. Recently, it was shown that optical spectroscopy in the visible and near-infrared ranges, when combined with ML models, enables non-destructive analysis of both DNA and GAG content, and prediction of overall construct maturity [182]. Besides the biochemical composition of the constructs, quantification of their mechanical properties is equally important for determining successful tissue functionality. Fibre-based fluorescence lifetime imaging has been used to non-destructively assess the biochemical composition of the engineered cartilage constructs, with algorithms developed to accurately correlate these optical measurements with their mechanical properties [183].

For the overall culture, feedback loops are important to evaluate the environment for temperature, oxygen, carbon dioxide, and signs of infection. To further optimize the culture and predict processes and outcomes in the bioreactor, *in silico* models could be useful. Especially when patient-specific cells or geometries in the final product are desired, digital twins are valuable. These are real-time virtual representations of physical counterparts (e.g. simulations that recreate patient-specific environments), which can help predict which parameters, including culture duration or mechanical stimulation, are optimal [65]. Furthermore, automated cleaning in a GMP-compliant manner is not typically discussed; however, it will limit the downtime of the system [67]. Examples of automated cleaning could be hydrogen peroxide gas with wipe disinfection, or using spray nozzles with decontamination products, and drying with sterilized air [67,75]. Alternatively, disposable components can be used for all parts that come into contact with cells during the production process.

## 6 Regulatory considerations

### 6.1 Framework for Advanced Therapy Medicinal Products

As of February 2025, only two Tissue Engineered Products (TEPs), that fall in the Advanced Therapy Medicinal Products (ATMPs) category, with active marketing authorization are approved by the EMA in Europe, namely Holoclar, approved in 2015, and Spherox, approved in 2017 [17]. Holoclar is a therapy developed for patients with damaged cornea and was the first approved ATMP using stem cells [184]. Spherox is a form of ACI, where cartilage defects are treated by seeding them with pre-cultured autologous AC microtissues [16,185,186]. The EMA classifies injected or implanted medicinal products used for treatments as ATMPs if they consist of manipulated tissues, cells, or genes [187]. For instance, ACI performed with autologous ACs that have only been isolated and washed, is not classified as an ATMP, as the cells are not manipulated. However, if the cells were expanded before implantation, and therefore manipulated, the therapy falls under the ATMP category. There is much interest in ATMPs, as they could offer a solution to diseases where other traditional therapeutic treatment strategies do not work sufficiently [188]. Also, they are widely investigated for their potential in personalized medicine, as they can be tailored to individual patients, sometimes using patient-specific material [189].

In order to obtain market authorization by the EMA, it is important that the efficacy, safety, and quality of the product are proven, and that the product is manufactured under GMP regulations and introduced to patients following good clinical practice [187,188]. The quality profile of an ATMP needs to be proven by a chemistry, manufacturing, and control (CMC) study, which shows that a standardized and controlled ATMP can consistently be achieved by using a validated manufacturing process. Simultaneously, safety should be assessed in non-clinical and clinical trials, especially during phase I [187]. Additionally, introducing process changes, such as the introduction of a novel automation technique, to an already regulatory approved process, could require extensive comparability and validation tests to verify that the changing process condition and methods did not cause product quality variations [65,67]. This highlights the importance of considering GMP methods, scalability, automation, standardization, quality controls, regulatory aspects, and cost-effectiveness early in the development phases of the manufacturing process of a TEP or an ATMP [65,67,72,75].

Similarly, regulatory considerations apply to the GMP compliance of bioinks used for bioprinted cartilage constructs to ensure safety, reproducibility, and regulatory acceptance [190]. Cell-laden bioinks are considered ATMPs, requiring donor eligibility verification, aseptic manufacturing strategies, and rigorous sterility control, including validated endotoxin testing [191]. The bioink regulations are complex due to the continuously evolving formulations and applications. They must have well-defined composition and specifications, with validated analytical methods to assess critical properties such as viscosity, pH, osmolality, and rheology [192]. Comprehensive documentation and traceability of all components and processes are also essential to meet regulatory requirements and ensure patient safety [193]. This contributes to the limited availability of GMP-compliant bioinks, creating a bottleneck in the adoption of bioprinting techniques for TEP production processes.

Next to the EMA, which was the first regulatory authority to include tissue engineered products in its framework in 2007, there are other regulatory organizations worldwide that manage these products [194]. For example, the FDA is responsible for regulatory decisions in the United States and the Pharmaceuticals and Medical Devices Agency (PMDA) in Japan. The assessment and classification of tissue engineered implants can vary across these agencies. The FDA classifies these products as human cell, tissue and cellular and tissue-based products (HCT/Ps). In contrast to the EMA, the FDA distinguishes the implants as either low-risk, middle-risk, or high-risk. A minimally processed product is classified in the low- and middle-risk category, and can reach the market quickly, since no premarket approval is required. High-risk categories, which commonly include tissue engineered products, do require a premarket approval, where the review process takes twelve months. By comparison, the EMA typically decides on the approval of a submission between nine and ten months [195]. The PMDA in Japan offers an additional accelerated pathway, called the conditional and time-limited early approval. To qualify, the safety and efficiency of the product for a disease where no successful treatment is available must be proven by the developers with clinical data [196,197]. This pathway, available since 2014, provides important opportunities to bring new innovations quickly to patients, and it saves money for manufacturers by avoiding long clinical trials [197]. However, when a product is not eligible for this shortened path, it can take up to seven years until approval [195]. To further accelerate the access of tissue engineered implants to patients worldwide, the field would benefit from international processes, requirements, and decision-making organizations. Beyond providing patients with suitable tissue engineered solutions, such alignment would also increase the manufacturing cost-effectiveness [194].

To assess the quality of the tissue-engineered construct automatically based on quantifiable data will further improve the cost-effectiveness of the system, as it decreases user differences or errors compared to time-consuming manual analysis [198]. However, using artificial intelligence (AI) for regenerative tissue engineering comes with additional regulatory obligations. AI-based systems should be transparent, meaning that the decisions made by the AI algorithm should be understandable. Furthermore, regulations must be taken into account regarding safety, training of the model, the privacy of the patient, and the accountability for decisions made by the system [199,200]. According to EMA guidance, a system risk management plan should be developed, particularly when human oversight is limited, to identify likely risks of failure modes of the algorithm. Additionally, if patient data are used during the manufacturing process, data privacy (General Data Protection Regulation, GDPR) and accountability must be ensured. Finally, when AI/ML-based systems influence the cartilage graft design or clinical application, they may be classified as medical devices and will necessitate Conformité Européenne (CE) marking in Europe [200].

### 6.2 Scalability and cost-effectiveness in a regulated setting

While developing a clinically functional tissue engineered implant, cost-effectiveness should be optimized to ensure its affordability. Two major drivers for costs within tissue engineering are personnel and the operation and certification of GMP-compliant facilities, such as clean rooms [67]. Using automated, closed systems with automatic cleaning can contribute to driving production costs down, as fewer personnel will be needed, while minimising the footprint of a necessary cleanroom facility. Continuous machine operation (24/7) can further increase the cost-effectiveness of the product, while automation also enhances robustness and reproducibility, as fewer human errors can occur, leading to more cost savings [142]. Using similarly sized disposables in the automated systems, such as culture flasks and tubes, also increases cost-effectivity as less time is needed for thorough cleaning and validation, especially when donor material is used [65].

Even automating a single step in the ATMP production process can significantly improve cost-effectiveness [123]. For example, iPSCs production is 42% less expensive by using the aforementioned StemCellFactory compared to manual production over an eight-year period, even though the investment costs for the automated system are doubled. The highest savings were attributed to the large decrease in staff costs [201]. When looking at other areas within tissue engineering, a fully closed and automatic procedure for making personalized skin grafts has been developed [65]. In this procedure, a biopsy from the patient is used to increase the size of the skin graft approximately 100 times in four weeks. The developed system consists of three compartments used for cell isolation from the biopsy, cell expansion, and skin formation [202]. Using this modularization in a bigger production process will likely also increase cost-effectiveness. In case a repair or update is needed, only a single module has to be replaced, allowing the rest of the system to remain intact. Furthermore, long-term maturation steps are inherently expensive, whereas these modular systems, where components could be exchanged for the production of the next tissue, may further decrease overall costs.

To ensure a cost-effective and efficient product, collaboration between several fields is essential, such as bioengineers, biologists, doctors, nurses, software engineers, and electrical engineers. Frequently overlooked, but highly important disciplines for the long-term stability of new TEPs are economics and policy regarding pricing and reimbursement [203]. Given that most ATMPs are often one-time procedures and carry high upfront costs, conventional payment models are insufficient to capture their long-term therapeutic and economic value. Additionally, the current reimbursement systems for ATMPs in Europe are challenging, with no uniform process across all countries [204]. Therefore, alternative reimbursement structures should be considered, such as outcome-based agreements and spread payments [205]. Economics and preparation of preclinical dossiers taking into account regulatory considerations should both be included early in the design process to evaluate cost-effectiveness and other socio-economic benefits, such as lower long-term health care costs and faster return to work, of the investment.

**Fig. 5:**
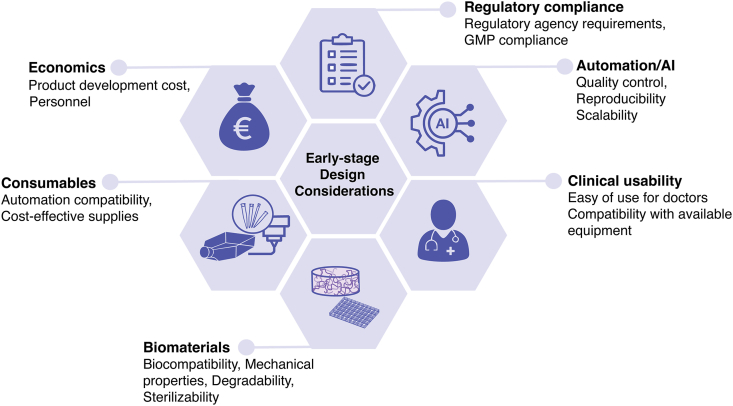
Summary of early-stage design considerations for the development of tissue-engineered cartilage implants. AI, Artificial Intelligence; GMP, good manufacturing practice.

The key factors that should be taken into consideration early in the design process of an engineered cartilage implant for successful, cost-effective, and regulation-compliant development are summarized in Fig. 5.

## 7 The future lab for cartilage tissue engineering

We envision the GMP-compliant production of cartilage tissue-engineered constructs for patients with cartilage defects in an automated and scalable manner. The automated system can be seen as a lab-in-a-box concept, where the entire process occurs in a closed-off box. This leads to an aseptic environment where lower International Organization for Standardization (ISO) norms are needed for the surroundings, or enabling the use of clean rooms of lower grade. The environment is continuously monitored and controlled, maintaining the O_2_ and CO_2_ concentration, the humidity, and the pH at predefined settings. The automated system should include automated cleaning to be operational at all times. Different compartments for different parts of the production process are needed, which can be assembled compactly. This concept allows the placement of such an automated system at the point of care, limiting logistical challenges and costs compared to current autologous tissue engineered implants produced at another location.

Regarding the compartments to add to the automated system, we envision that it supports the cryo-storage for off-the-shelf cells and the isolation of autologous cells. Suspension cultures can be used to expand and mature the cells and produce the microtissues in a scalable way. The medium changes in suspension cultures can be automated via tubing. After microtissue production in the suspension culture, the microtissues should be evaluated for quality based on cell phenotype and ECM content, and harvested. Including a compartment for the cryo-storage of the microtissues made by off-the-shelf or allogenic cells adds value by enhancing the cost-effectiveness of the process [206]. To have the benefits of both bottom-up and top-down tissue engineering, combined in a middle-out approach, the microtissues should be printed in a 3D printed scaffold based on the geometry of the patient. Bioreactors that achieve compression and shear stresses should be used to further mature this construct. Non-invasive quality checks, such as real-time assessment of microtissue morphology and aggregation with brightfield microscopy or Raman spectroscopy for evaluation of construct maturation, should result in an AI feedback loop that adjusts the culture based on the needs for maturation and growth of the cells and micro- and macrotissues. Reinforcement learning, which is commonly used in robotic-based systems, can be used to let the system make decisions based on the data from the environment and quality checks to optimize the end product [198,207]. Robot arms able to manipulate culture hardware can facilitate moving the cells or constructs between different compartments. It is of great significance that the platform should be easily adaptable to integrate new technologies. Using a modular system with interchangeable compartments allows for the seamless integration of these new technologies or repairs when needed.

## 8 Conclusion

This review highlights that cartilage microtissues have gained tremendous attention as modular components for building larger, functional engineered cartilage implants due to their ability to better mimic native tissue organization compared to single-cell approaches. Furthermore, microtissues are easily incorporated into new automated and scalable manufacturing techniques, enhancing their clinical translation potential. However, as the low number of tissue engineered implants approved by regulatory agencies shows, significant challenges remain in achieving scalable manufacturing and fully integrated, end-to-end manufacturing workflows. Comparative studies that evaluate different cell sources combined with various assembly techniques to determine suitable combinations for GMP-compliant and scalable tissue engineering are scarce. Addressing the key limitation of incomplete microtissue remodelling into a coherent and mechanically stable tissue requires research to identify optimal microtissue sizes and maturation protocols prior to fusion, and effective post-fusion maturation procedures. Furthermore, the development of standardized and approved processes is hindered by available manufacturing materials. Fortunately, increased awareness of the need for compliant bioinks and bioprinters is a great development and may help solve the current bottlenecks in the pathway for approval of new tissue engineered implants. Moving forward, the standardization of complete production processes will require the collaboration between different professions, where all aspects from tissue production to AI based process monitoring and quality control with automatic cleaning are optimized. A focus should be given on implementing and combining GMP-compliant, automated, scalable, socio-economical, and regulatory concepts early in the design process. Ultimately, this should bring us closer to cost-effective and scalable tissue engineering solutions to create ready-to-implant cartilage constructs for patients.

## Declaration of Competing Interests

The author(s) have no conflicts of interest relevant to this article.

## Author contributions

DMAM: conceptualization, investigation, project administration, writing - original draft, and writing - review & editing.

AGV: visualization, investigation, project administration, writing - original draft, and writing - review & editing.

FA: conceptualization, supervision, writing - review & editing.

JVK: supervision, writing - review & editing.

AKÖ: supervision, writing - review & editing.

IP: writing - review & editing.

DJK: writing - review & editing.

SJPC: conceptualization, supervision, writing - review & editing.

JM: supervision, writing - review & editing.

KI: conceptualization, supervision, funding acquisition, writing - review & editing.

The author(s) have no conflicts of interest relevant to this article.

## Declaration of generative AI and AI-assisted technologies in the manuscript preparation process

During the preparation of this work, the authors used Copilot in order to improve the readability of the text. After using this tool, the authors reviewed and edited the content as needed and take full responsibility for the content of the published article.

## Funding

This publication is part of the project LS-CarE (with project number NWA.1389.20.192) of the research program NWA-ORC which is (partly) financed by the Dutch Research Council (NWO). The authors would like to kindly acknowledge the financial support from the Gravitation Program “Materials Driven Regeneration” (024.003.013), the Summit Program “DRIVE-RM” (SUMMIT.1.027), and the TTW Veni 2024 Program (21866), all funded by The 10.13039/501100003246Dutch Research Council (10.13039/501100003246NWO). 10.13039/100014337Furthermore, this project has received funding from the 10.13039/501100000781European Research Council (ERC) under the European Union's 10.13039/100018693Horizon Europe research and innovation program (Re-COLL; grant agreement No [101142063]) and the European Union's 10.13039/100018693Horizon Europe research and innovation program (m2M; grant agreement No [101191729]).
